# Probing and predicting ganglion cell responses to smooth electrical stimulation in healthy and blind mouse retina

**DOI:** 10.1038/s41598-020-61899-y

**Published:** 2020-03-23

**Authors:** Larissa Höfling, Jonathan Oesterle, Philipp Berens, Günther Zeck

**Affiliations:** 1 Natural and Medical Sciences Institute at the University of Tübingen, Reutlingen, Germany; 2 Graduate Training Centre of Neuroscience, University of Tübingen, Tübingen, Germany; 3 Institute for Ophthalmic Research, University of Tübingen, Tübingen, Germany; 4 Centre for Integrative Neuroscience, University of Tübingen, Tübingen, Germany; 5 Bernstein Center for Computational Neuroscience, University of Tübingen, Tübingen, Germany; 6 Department of Computer Science, University of Tübingen, Tübingen, Germany

**Keywords:** Extracellular recording, Retina

## Abstract

Retinal implants are used to replace lost photoreceptors in blind patients suffering from retinopathies such as retinitis pigmentosa. Patients wearing implants regain some rudimentary visual function. However, it is severely limited compared to normal vision because non-physiological stimulation strategies fail to selectively activate different retinal pathways at sufficient spatial and temporal resolution. The development of improved stimulation strategies is rendered difficult by the large space of potential stimuli. Here we systematically explore a subspace of potential stimuli by electrically stimulating healthy and blind mouse retina in epiretinal configuration using smooth Gaussian white noise delivered by a high-density CMOS-based microelectrode array. We identify linear filters of retinal ganglion cells (RGCs) by fitting a linear-nonlinear-Poisson (LNP) model. Our stimulus evokes spatially and temporally confined spiking responses in RGC which are accurately predicted by the LNP model. Furthermore, we find diverse shapes of linear filters in the linear stage of the model, suggesting diverse preferred electrical stimuli of RGCs. The linear filter base identified by our approach could provide a starting point of a model-guided search for improved stimuli for retinal prosthetics.

## Introduction

Sensory neural prostheses attempt to replace nonfunctional elements of sensory organs and thereby to restore access to sensory information. For example, cochlear implants, which have been in clinical use for many years, replace the sensory receptors of the auditory system and can significantly improve auditory function and quality of life of implanted patients^1^. Likewise, retinal implants aim to replace the function of the sensory receptors of the visual system, the photoreceptors, in retinopathies such as retinitis pigmentosa. While there are different approaches to retinal implants in terms of implantation site and technical realizations (subretinal^2–4^; epiretinal^5^; suprachoroidal^6^), all use short rectangular pulsatile stimuli to activate the remaining retinal neurons. Patients implanted with a retinal prosthesis experience benefits in mobility and navigation and some regain rudimentary but useful visual functions like the capability to identify, localize and discriminate objects^2,7^.

Despite this success, there are a number of shortcomings in implant-aided vision, many of which are due to the fact that the electrical stimuli are very different from the physiological signals in the retina, which are much slower and smoother than the pulsatile electrical stimuli^8–12^. Incidental activation of axons passing the stimulation target (e.g. a single retinal ganglion cell (RGC)) severely limits spatial resolution^13–16^, while desensitization of retinal neurons to repetitive electrical stimulation impedes high temporal resolution^17–19^. Acuity of prosthesis-mediated vision therefore reaches only a fraction of healthy visual acuity, leaving patients legally blind despite wearing a retinal implant^20^. Another reason why bionic vision comes short of natural vision is that the image processing mechanisms of the intricate retinal network cannot be triggered selectively by retinal implants; pulsatile stimuli do not separately activate channels like the ON and the OFF pathway^21,22^.

Different approaches have been suggested to tackle these problems. Placing the stimulation electrodes in different positions within the retina and varying the pulse durations confers control over which retinal elements are activated by pulsatile electrical stimulation^23^. The activation of axons of passage can be avoided by increasing the stimulus duration, thus improving spatial resolution of implant-aided vision^14^. Varying the shape of the stimulation waveform can increase the charge-efficiency^24^ and the selectivity of the stimulus with respect to cell type and mode of activation^23,25^. Cells in the different layers of the rabbit retina preferentially respond to increasing frequencies of sinusoidal stimulation as one moves from the outer nuclear to the ganglion cell layer^26^. ON and OFF RGCs respond in different phases of low-frequency sinusoidal stimulation; however this phase-preference was assigned to activation of photoreceptors. The clinical relevance of sinusoidal stimulation thus appears to be limited^25^. Varying stimulus parameters such as shape, repetition rate, duration and charge allowed to identify stimulus regimes that maximize the response ratio of ON and OFF RGCs^27–31^.

However, the insight gained by such heuristic searches for optimal stimulus parameters is limited to a small area of the parameter space. One way to more exhaustively look for optimal stimuli for a given sensory modality is white noise analysis^32–34^. This concept has been successfully applied to stimulation with the natural stimulus modality in different sensory systems, such as visual stimulation in the visual system^35^, whisker deflection in the vibrissal system in rats^36^, but also to artificial, electrical stimulation in the visual system^33,37^. Recently, white noise composed of biphasic electrical current pulses with amplitudes drawn from a Gaussian distribution was used to map the spatiotemporal electrical receptive fields of rat retinal ganglion cells. Subsequently fit linear-nonlinear models accurately predicted RGC responses to electrical stimulation^38,39^. Linear filters of mouse RGCs could also be recovered with a spatially uniform white noise stimulus consisting of normally distributed subthreshold voltage pulses^40,41^. The results of these studies demonstrate the feasibility of the white noise analysis approach in electrical stimulation. However, these studies sampled subspaces of the stimulus space, varying pulse amplitude, but not duration or frequency. As white noise analysis can only identify the stimulus that is optimal within the space of provided stimuli, one should try to sample a space that is most likely to contain the true optimal stimulus.

Therefore, we developed and applied a smooth electrical Gaussian white noise current stimulus to cover a space of stimuli that more closely approximate the time scale of physiological signals in the retina. This smooth stimulus simultaneously probes the preferences of RGCs with respect to amplitude, polarity and frequency of an electrical stimulus. Using a high-density CMOS-based microelectrode array for stimulation, we were able to reliably activate RGCs in both wild-type mouse retina (*wt*) and retina from the *rd10* mouse model of retinal degeneration. We estimated linear filters of cells using two approaches to fitting a linear-nonlinear-Poisson (LNP) model: spike-triggered averaging (STA) and maximum likelihood estimation (MLE). Probing the light responses of RGCs in *wt* retina allowed us to relate electrical filter shapes to light response profiles. The LNP model accurately predicted RGC responses to electrical stimulation, demonstrating that it captures aspects of retinal processing of electrical stimuli relevant for response generation. The model may be useful for guiding the search for stimuli that improve spatial and temporal resolution of prosthetic-aided vision. The linear filters described here provide a starting point for this search.

## Results

### Simultaneous electrical stimulation and recording using a high-density CMOS-MEA

**Figure 1:**
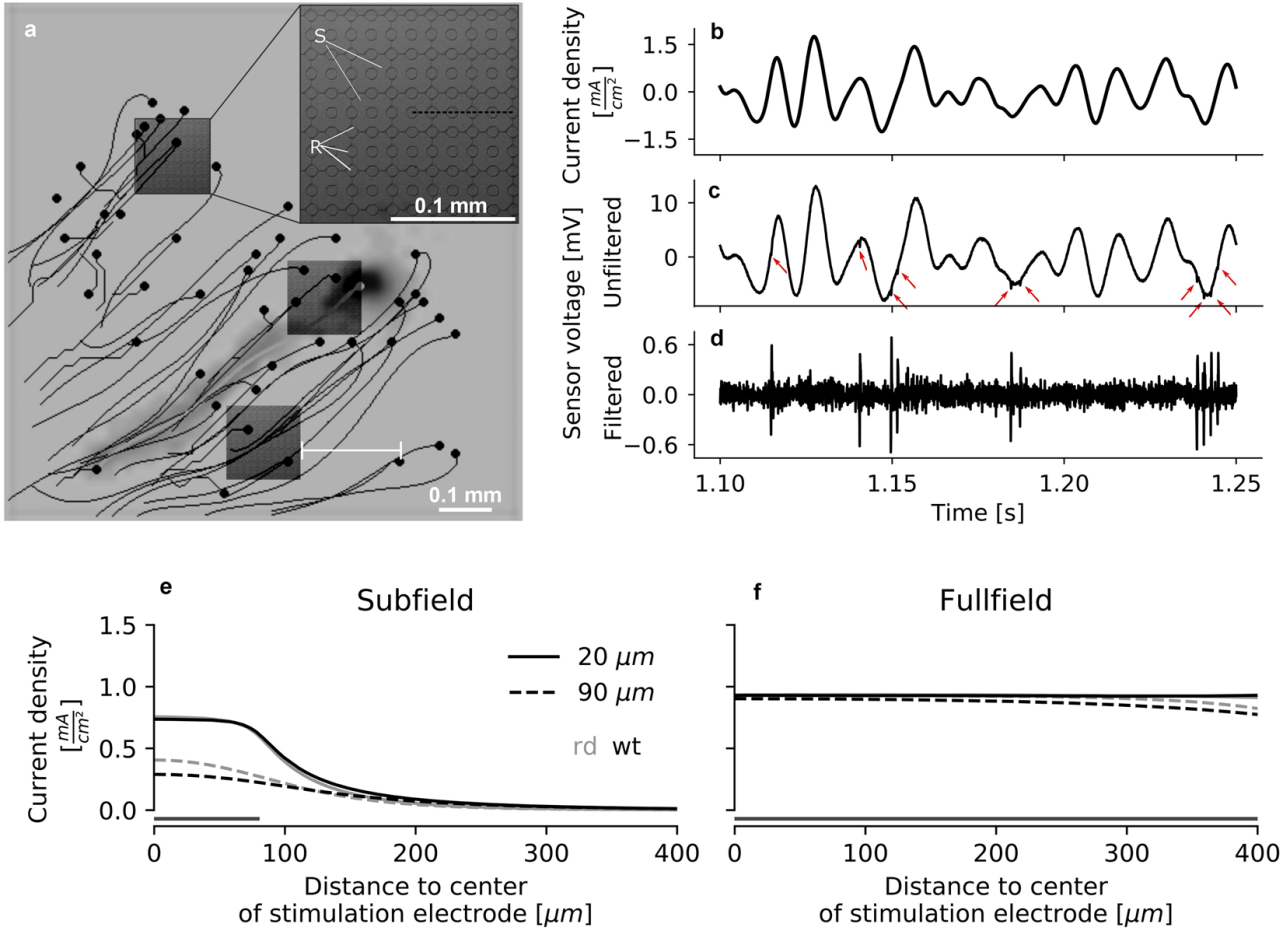
Simultaneous stimulation and recording of RGCs using a hd CMOS-MEA (**a**) Schematic of soma location (black dots) and axon traces (black lines) of RGCs as measured by the stimulation and recording software, and configuration of active stimulation areas (dark gray squares) in one example recording. The retina (here from *bl6* mouse) was flat-mounted on a hd CMOS-MEA (gray background). The horizontal white line illustrates how the distance of a cell’s soma from the closest stimulation area was determined. The inset shows the grid of stimulation electrodes (large elements, labelled S) and recording electrodes (small elements, labelled R). In most recordings, only a subset of the stimulation electrodes were active (i.e. delivering the stimulation current). The black dashed line indicates the path from the center of the stimulation area to the edge along which current density was simulated (see panel (e)). Cell activity was recorded simultaneously on recording electrodes. (**b**) Expected current density of the smooth electrical Gaussian white noise stimulus, calculated as the derivative of the voltage command (see Methods, Eq. (1)). (**c**) Raw recording signal upon stimulation with the stimulus shown in (**b**). The stimulus causes an artefact in the raw recording orders of magnitude larger than the signal of interest, the spikes, indicated by red arrows. (**d**) Signal after filtering with a ${2}^{nd}$ order band-pass Bessel filter between 1000 and 9500 Hz and artefact subtraction. The artefact is removed from the signal and spikes are clearly detectable. (**e**,**f**) Simulation of the current density at different heights above the stimulation electrodes in *wt* (black) and *rd10* (gray) retina in the subfield and fullfield condition, respectively (see Methods). The solid lines represent simulated current at a height of 20 $\mu m$, corresponding to the ganglion cell layer^67,68^, and dashed lines represent simulated current at a height of 90 $\mu m$, approximately corresponding to the inner nuclear layer. The extent of the stimulation area is indicated by the line parallel to the x-axis. The distance of the path from the center to the edge was measured at a 0${}^{\circ }$ angle (dashed black line in inset in panel (a)).

We used a smooth electrical current stimulus applied by a high-density CMOS-microelectrode-array (hdCMOS-MEA 5000) to stimulate *ex-vivo* flatmount preparations of wild-type and photoreceptor-degenerated retina in epiretinal configuration. Our setup allowed us to simultaneously and continuously electrically stimulate on an arbitrary subset of the 1024 stimulation electrodes and record on 4225 recording electrodes (Fig. 1a,e,f). After the smooth stimulation waveform was removed from the recording, spike-sorting allowed to analyse the retinal ganglion cell responses to the stimulus at the level of individual cells (Fig. 1b–d). We evaluated the retinal response in wild-type retina (n = 3, *wt*) and blind retina (n = 5, *rd10*) to electrical stimulation by computing the temporal linear electrical filters of the RGCs in a model-based approach.

### Reliable RGC responses to smooth electrical Gaussian white noise stimulation

**Figure 2:**
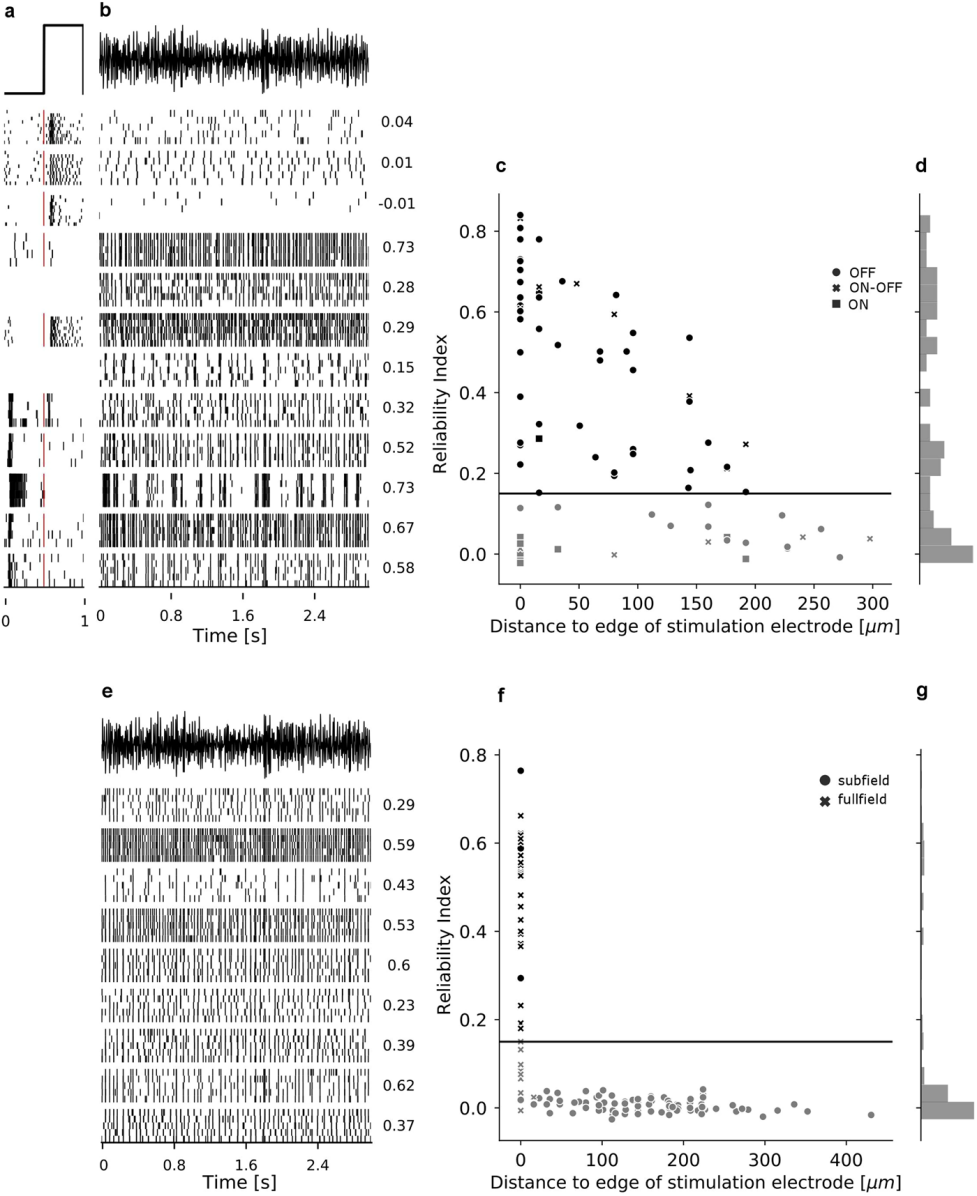
RGC responses to light flashes and smooth electrical stimulation. (**a**) Raster plots of the responses of RGCs from *wt* mouse retina to a fullfield light flash stimulus. The time course of the stimulus is indicated in the first row, and light onset is marked by a red vertical line in every raster plot. Not every cell was recorded both during electrical and light stimulation. (**b**) Raster plots of the responses of RGCs from *wt* mouse retina to an excerpt from the smooth electrical Gaussian white noise stimulus (stimulus shown in the first row). Numbers next to each row indicate the reliability index *RI* of the cell’s response to electrical stimulation. (**c**) Reliability Index as a criterion for reliability of the response is plotted against distance to the edge of the closest active stimulation electrode. Black horizontal line indicates reliability threshold $RI > 0.15$ for inclusion in further analysis. Different markers indicate ON, ON-OFF and OFF cells, determined by their Bias Index as described in the Methods section. (**d**) Histogram of the reliability indices of *wt* RGCs. (**e**) to (**g**) same as (**b**) to (**d**) but for RGCs from *rd10* retina. Different markers in (**f**) indicate cells recorded in the subfield and fullfield condition (see Methods).

Retinal ganglion cells in healthy and blind mouse retina responded reliably to stimulation with smooth electrical Gaussian white noise (Fig. 2b,e). For some cells, firing patterns were nearly identical across trials (Fig. 2b, fourth and fifth row (*wt*); and e, second row from below (*rd10*)); others were more variable in their response (Fig. 2b, fifth row from below (*wt*); and e, third row (*rd10*)). We quantified the reliability of the RGC response to the electrical stimulus by computing a *reliability index, RI* (see Methods). In *wt* retina, the majority of RGCs (N = 53/84, 63%) were entrained to the stimulus with a *RI* larger than a threshold of 0.15 (Fig. 2d). In *rd10* retina, a smaller percentage of cells were above threshold (N = 26/126, RI $>$ 0.15, Fig. 2g); however, the levels of reliability among above-threshold cells were comparable between *rd10* and *wt* retina. A simulation of lateral and vertical current spread, taking into account different retinal thickness in *wt* and *rd10* retina, showed that the effect of this difference in thickness on stimulation intensity was negligible (see Fig. 1e,f).

One major challenge in bionic vision is the selective activation of different information channels such as the ON and the OFF channel^42,43^. In order to be able to evaluate whether RGCs belonging to these retinal processing pathways are differently affected by our stimulus, we classified RGCs from healthy retina as ON, OFF or ON-OFF based on their light response. We presented light flashes (0.5 s OFF, 0.5 s ON; see Methods and Fig. 2a) and recorded the RGC responses to light stimulation. Of the cells that could be identified across recordings with light and electrical stimulation (47/84), 10 cells increased their spiking during incremental light stimuli (ON response, Fig. 2a, rows 1-3, row 6), 23 increased their activity during light decrements (OFF response, Fig. 2a, row 4, last five rows), and the remaining 14 cells had no clear preference for ON or OFF stimuli (ON-OFF or unknown, Fig. 2a, row 5). Only one of the 10 cells with ON response passed the reliability criterion of $RI > 0.15$ during electrical stimulation (see Fig. 2a,b, row 6), while 19/23 cells with OFF response and 8/14 cells with ON-OFF response responded reliably to electrical stimulation.

The reliability of the RGC response demonstrates that smooth electrical Gaussian white noise can efficiently activate RGCs in healthy and blind mouse retina. The different percentages of activated cells in *wt* and *rd10* retina can be explained by a larger spatial extent of the sensitivity to electrical stimulation in *wt* compared to *rd10* retina.

### Localized response to local electrical stimulation

Previous studies have investigated the spatial extent of the *electrical receptive field* (spatial eRF), i.e. the area in which electrical stimulation evokes a response in a cell. In healthy rat retinas, eRF with diameters of about $250\,\mu m$ have been reported, while the eRF in blind rat retinas was found to span about $200\,\mu m$ in diameter^4^. Recent studies in healthy and blind mouse retina found eRF diameters of around $400\,\mu m$ for RGCs in healthy mouse retina, and diameters of around $350\,\mu m$ for RGCs in blind mouse retina^44,45^. These eRF sizes were found upon network-mediated activation of RGCs in response to subretinal stimulation with pulsatile stimuli. A different study investigated how the activation threshold of rabbit RGCs depends on the distance between the RGC and the stimulation electrode upon epiretinal electrical stimulation^46^. The threshold was found to increase with distance; this increase was much steeper for direct activation compared to network-mediated activation.

Here, we investigated the spatial extent of the sensitivity of RGCs to smooth electrical stimulation applied epiretinally in *wt* and *rd10* retina by stimulating subfields of the electrode array and analyzing how RGC responses vary with distance from the stimulation areas. A simulation of the lateral current spread at different heights above the active stimulation electrodes shows that the stimulation in the subfield condition is indeed locally confined (Fig. 1e; see Methods). Simulated current density drops with distance from the center of the stimulation area; it does so more steeply at the level of the RGCs compared to the level of the INL. Simulated stimulation intensity is comparable between subfield and fullfield condition at the RGC level, whereas it is stronger in the fullfield condition at the INL level (Fig. 1e,f).

We found that the reliability of the RGC response to the electrical stimulation decreased with increasing distance from the closest active stimulation electrode (Fig. 2c,f). In *wt* retina, the majority of cells within a radius of $200\,\mu m$ of an active stimulation electrode were entrained to the stimulus ($RI > 0.15$). Cells outside this radius did not respond robustly to the stimulus ($RI\le 0.15$). This is largely in agreement with eRF sizes reported previously in studies using subretinal pulsatile stimulation. We hypothesized that the small fraction of ON cells responding to electrical stimulation was due to the fact that by chance, the recorded ON cells were located further away from active stimulation electrodes. However, this was not the case; ON cells were recorded at distances from $0$ to $\approx 200\,\mu m$ from the closest active stimulation electrode (Fig. 2c).

In contrast, RGCs from *rd10* retina responded only in the fullfield condition (Fig. 2f, crosses), or if they were located directly adjacent to or above an active stimulation area in the subfield condition (Fig. 2f, dots; see Methods). Specifically, 23 of 35 (65.7%) RGCs from *rd10* retina responded reliably in the fullfield condition, while 3 of 4 cells located at zero distance from the stimulation electrodes in the subfield condition responded reliably to electrical stimulation. RGCs located further away from the stimulation electrodes did not respond to the stimulus (total number of cells in subfield condition: 91). Thus, the spatial extent of the effect of our stimulus is very localized in *rd10* retina and more spread out in *wt* retina. This difference in spatial extent suggests that different mechanisms underlie the RGC response to smooth electrical stimulation in healthy compared to blind mouse retina.

### Temporal electrical linear filters of RGCs

**Figure 3:**
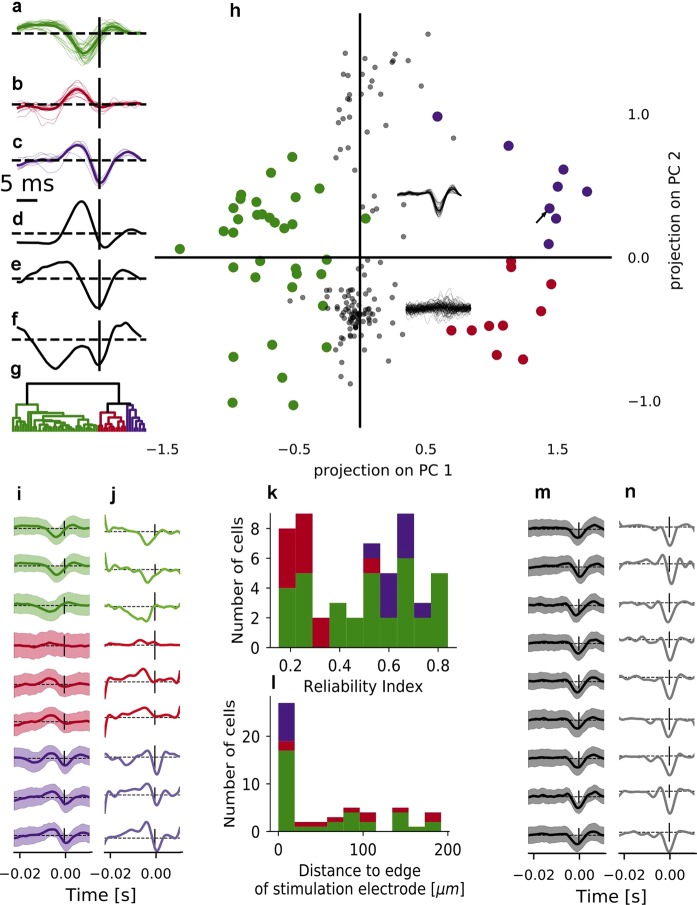
Hierarchical clustering of RGC electrical temporal filters estimated by STA. (**a**–**c**) Electrical temporal filters of all reliably responding RGCs from *wt* retina recovered from the STA fit of the LNP model, displayed separately for the three clusters identified by the hierarchical clustering algorithm. Thin lines are individual cell filters, thick lines indicate the average filter for one cluster. (**d**,**e**,**f**) 1st, 2nd and 3rd principal component (PC) recovered from principal component analysis of the ensemble of temporal filters from all cells. (**g**) Dendrogram showing the separation of consecutively joined clusters along the clustering metric (distance in euclidean space). (**h**) Scatter plot of the projections of the temporal filters onto the 1st and 2nd PCs (shown in (**d**,**e**)). Colors indicate cluster assignment. Grey dots indicate the projections of filters of RGCs from *rd10* retina, projected onto the same PCs. The lower inset shows the filters of all *rd10* RGCs whose filter projections are negative in PC2; the upper inset shows the filters of all *rd10* RGCs whose filter projections are positive in PC2. The black arrow marks the cell for which the assignment to clusters did not agree between STA and MLE estimate of the filters. (**i**) STA estimates of the filters of example RGCs from *wt* retina shown in Fig. 2b (rows 4-12, same order), with cluster assignment indicated by color. (**j**) Same as (**i**), but for MLE estimates of the filters. (**k**) Stacked histogram of the distribution of reliability indices (RIs) for all cells with $RI > 0.15$, color-coded according to cluster assignment. (**l**) Distribution of distance to the edge of the closest active stimulation area of all responsive cells ($RI > 0.15$), color-coded according to cluster assignment. (**m**) STA estimates of the filters of example RGCs from *rd10* retina shown in Fig. 2e (same order). (**n**) Same as (**m**), but for MLE estimates of the filters.

We computed temporal electrical linear filters by fitting a linear-nonlinear-Poisson model using two different methods, spike-triggered averaging (STA) and maximum-likelihood-estimation (MLE). We did this for all cells which responded reliably to the stimulus (reliability index $RI > 0.15$, N = 53/84 in *wt* retina, N = 26/126 in *rd10* retina). While filters of RGCs from *rd10* retina were all monophasic negative (Fig. 3m,n), filter shapes of RGCs from *wt* retina were more variable (Fig. 3i,j).

**Figure 4:**
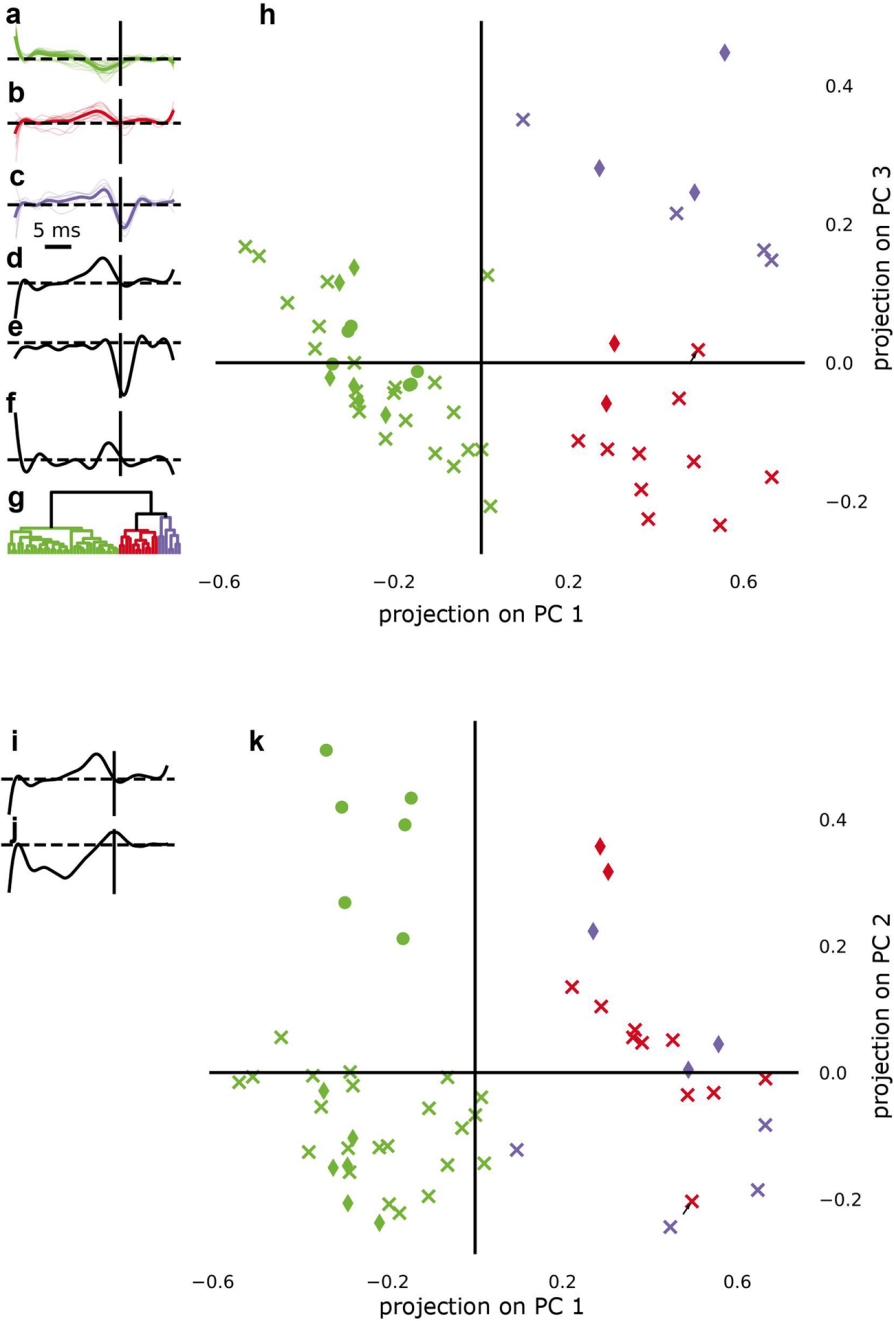
Hierarchical clustering of RGC electrical temporal filters estimated by MLE. (**a**–**c**) Electrical temporal filters of all RGCs from wild-type retina recovered from the MLE fit of the LNP model, displayed separately for the three clusters identified by the hierarchical clustering algorithm. Thin lines are individual cell filters, thick lines indicate the average filter for one cluster. (**d**,**e**,**f**) 1st, 3rd and 4th principal component (PC) recovered from principal component analysis of the ensemble of temporal filters from all cells. (**g**) Dendrogram showing the separation of consecutively joined clusters along the clustering metric (distance in euclidean space). (**h**) Scatter plot of the projections of the temporal filters onto the 1st and 3rd PCs (shown in (**d**,**e**)). Colors indicate cluster assignment. Different markers (filled circle, cross and diamond) indicate cells recorded in different sessions. The black arrow marks the cell for which the assignment to clusters did not agree between STA and MLE estimate of the filters. (**i**,**j**) 1st and 2nd PC recovered from principal component analysis of the ensemble of temporal filters from all cells. (**k**) Scatter plot of the projections of the temporal filters onto the 1st and 2nd PCs (shown in (**i**,**j**)). Colors indicate cluster assignment. Different markers (filled circle, cross and diamond) indicate cells recorded in different sessions. Note that the 2nd PC separates filter projections of cells from different recordings, but does not separate the red and the violet cluster.

To quantify the difference between the filter shapes found in wild-type retina, we performed a hierarchical clustering on the projection of the filters onto three principal components (PC). For STA filters, the first three PCs (3d–f) jointly explained 95% of the variance (PC1: 72.1%, PC2: 18.1%, PC3: 5.1%). For the MLE filters, the first five PCs were needed to explain 95% of the variance; we projected onto PC1, PC3 and PC4, explaining 69.1%, 9% and 3% variance, respectively (Fig. 4d,e,f), leaving out PC2. We did this because the variance explained by PC2 (12.4%) was mostly due to variance between different recordings, which was not of interest here (Fig. 4i–k).

We identified two distinct clusters for both STA and MLE filters based on the dendrogram generated by the hierarchical clustering algorithm (see Figs. 3g and 4g). These two clusters were separated along the axis of the first PC, and one of them could be split into two sub-clusters that were separated along the axis of the second (STA) or third (MLE) PC. The clusters corresponded to three filter shapes, monophasic negative (green, Figs. 3 and 4a), monophasic positive (red, Figs. 3 and 4b) and biphasic (violet, Figs. 3 and 4c). Assignment to clusters agreed between the STA and the MLE filter estimates for all except one cell. The estimates of the filters of the reliably responding example cells from *wt* retina (Fig. 2b rows 4–12) fall into these three categories (Fig. 3i,j). Filters from *rd10* retina were all monophasic negative (Fig. 3m,n, using the same example cells as in Fig. 2e). The acausal components observed in some filters are likely due to remaining correlations in the stimulus that could not be entirely removed by the whitening procedure (see Methods and Supplementary Fig. 1).

In order to compare the filters of RGCs from *wt* and *rd10* retina, we projected the LNP estimates of the filters from *rd10* RGCs (Fig. 3g, gray dots) onto the LNP PCs obtained from *wt* retina. The densely grouped projections in the lower part of the plot corresponds to projections of flat filters from non-responsive cells, while the more spread-out upper group corresponds to projections of filters from responsive cells (Fig. 3g, insets). Visual comparison of the filter shapes suggests that, while filters from *rd10* RGCs do not fall into one of the three clusters found in *wt*, they are more similar to monophasic negative and biphasic than to monophasic positive *wt* filters (Fig. 3i,j,m,n). The pattern of clustering described above confirms this observation.

We calculated the latencies of the (positive and/or negative) peaks of the STA and MLE filters of *wt* and *rd10* RGCs relative to the spike (see table 1). In *wt* retina, the latencies of the peaks of monophasic positive filters were around 2 ms longer than the latencies of the peaks of monophasic negative filters. For cells with biphasic filters, the spike occurred within 1 ms of the negative peak of the filter, preceded by 3 to 8 ms by the positive peak. For the monophasic negative filters of RGCs from *rd* retina, the spike occurred within less than 2 ms of the peak of the filter, comparable to the negative peak latencies observed for biphasic filters in *wt* retina.

**Table 1:** Peak latencies of filters The median and the range of the peak latencies relative to spike time for STA filters (top) and MLE filters (bottom). Negative values indicate that the peak occurred before the spike.

	latency of neg. peak [ms]	latency of pos. peak [ms]
monophasic neg. (green, N = 35)	$-3.7,[-6.1,-1.3]$	
	$$-3.3,[-5.4,0.2]$$	
monophasic pos. (red, N = 11)		$-5.5,[-6.2,-4.1]$
		$$-4.7,\hspace{2.22144pt}\hspace{2.22144pt}\hspace{2.22144pt}[-6.6,-3.2]$$
biphasic (violet, N = 9)	$\hspace{2.22144pt}\hspace{2.22144pt}\hspace{2.22144pt}0.2,[-0.1,0.7]$	$-5.2,[-7.7,-4.5]$
	$-0.3,[-0.9,0.2]$	$-3.1,[-7.3,-2.9]$
*rd10* (N = 28)	$-0.6,[-1.2,0.3]$	
	$$0.0,[-0.6,0.8]$$	

### Electrical linear filters vary with light response profiles of RGCs

**Figure 5:**
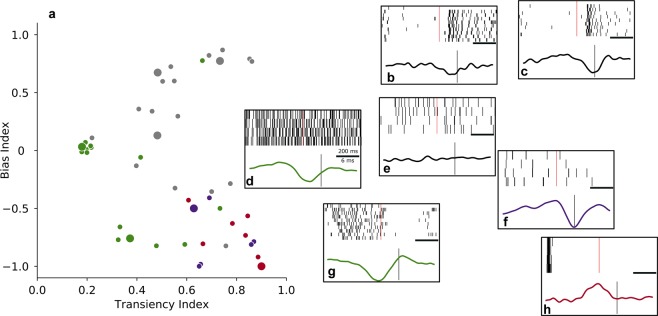
Electrical linear filters vary with light response profiles of RGCs. (**a**) Distribution of cells according to their light response profile. Color codes for cluster assignment of a cell based on its electrical linear filter. Grey dots indicate cells which were not included in the clustering due to below-threshold response reliability in the electrical stimulation condition ($RI < 0.15$). Bias Index quantifies the polarity of the light response from OFF (−1) through ON-OFF (around 0) to ON (1); Transiency Index quantifies duration of the response to a cell’s preferred light stimulus from sustained (0) to transient (1). (**b**–**h**) Example cells with different light response profiles, indicated in (**a**) by larger dots. Upper plot in each panel shows a raster plot of the cell’s light response in 4–10 repetitions of the light step stimulus. Light onset is indicated by a vertical red line. Lower plot in each panel shows the cell’s electrical linear filter as estimated by STA in the LNP model; color coding as in panel a. Vertical line indicates spike time. The timescale bar corresponds to 200 ms in the upper plot, and to 6 ms in the lower plot.

Different RGC types, such as ON and OFF or sustained and transient types, constitute different information channels sending a preprocessed version of the visual scene to the brain^43,47^. Preserving this first decomposition of the visual input into different information channels by separately activating RGCs of different types would significantly improve implant-aided vision^48^. We therefore investigated whether there was a relationship between the light response profiles of RGCs from *wt* retina and their electrical linear filters. We characterized RGCs in terms of their preference for light polarity (ON, ON-OFF or OFF), and in terms of their response duration (transient, intermediate or sustained) by calculating a Bias Index and a Transiency Index (see Methods). We found transient and intermediate OFF and ON cells as well as sustained and intermediate ON-OFF cells (Fig. 5a). Transient OFF cells had biphasic or monophasic positive filters (Fig. 5f,h), while intermediate OFF cells and sustained ON-OFF cells had monophasic negative filters (Fig. 5g,d). Most ON cells did not respond reliably to the electrical stimulus ($RI < 0.15$) and could therefore not be assigned to a cluster based on their electrical linear filter. Electrical linear filters of most ON cells were rather noisy (Fig. 5b,e) due to their poor response to the stimulus; however, some ON cells had filters with a discernible negative deflection at spike time (Fig. 5c). The observed pattern suggests a difference in preferred electrical stimulus between RGCs with transient and sustained light responses.

### LNP model accurately predicts RGC responses to electrical stimulation

**Figure 6:**
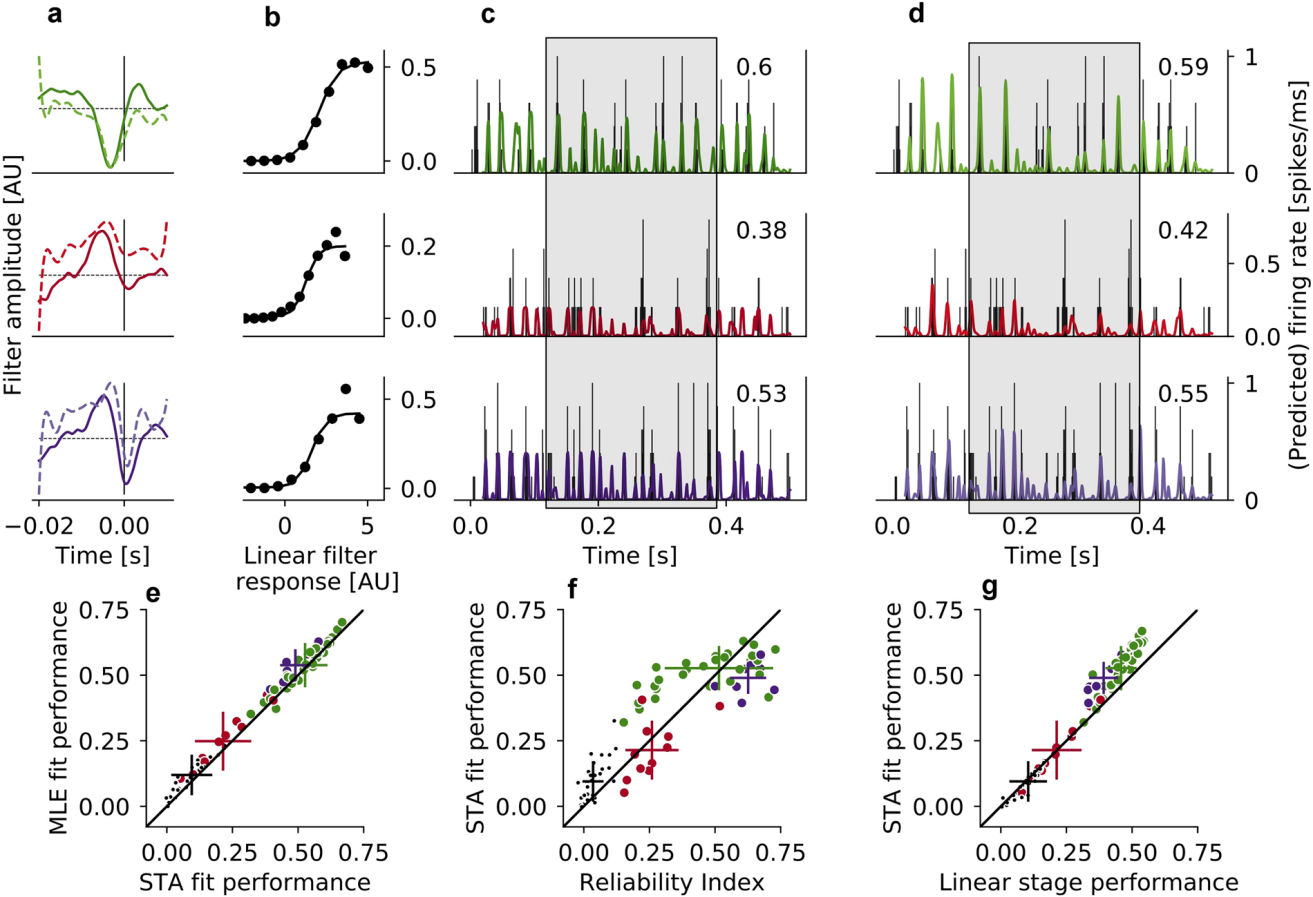
Model prediction of firing rates in *wt* retina. (**a**) Linear filters of three example cells from different clusters, derived from the STA fit (solid lines) and from the MLE fit (dashed lines); displayed at different scales in arbitrary units. To create the firing rate prediction, the filter response of the stimulus snippets with these filters was computed, and then the nonlinearity was applied. (**b**) Nonlinearity of the STA model; the nonlinearity of the MLE was the standard sigmoid function. (**c**) True firing rate, binned into 1 ms bins (black histogram) and the firing rate prediction of the STA fit of the model (colored traces) for three example cells; the highlighted region shows differential responses of cells with different types of filters, which are correctly predicted by the model. Decimal numbers in upper right corner indicate the performance of the model for that specific cell. (**d**) Same as (**c**) for the MLE fit of the model. (**e**) Prediction performance of the MLE fit plotted against the prediction performance of the STA fit; colors indicate cluster assignment. Small black dots represent RGCs that did not pass the reliability criterion of *RI* $> \,0.15$, but are shown here for completeness. (**f**) Prediction performance of the STA fit plotted against reliability index. Colors indicate clusters assignment. Small black dots as in (**e**). (**g**) Prediction performance of the STA fit of the full LNP model plotted against the prediction performance of only the linear stage of the LNP model. Colors indicate cluster assignment. Small black dots as in (**e**).

**Figure 7:**
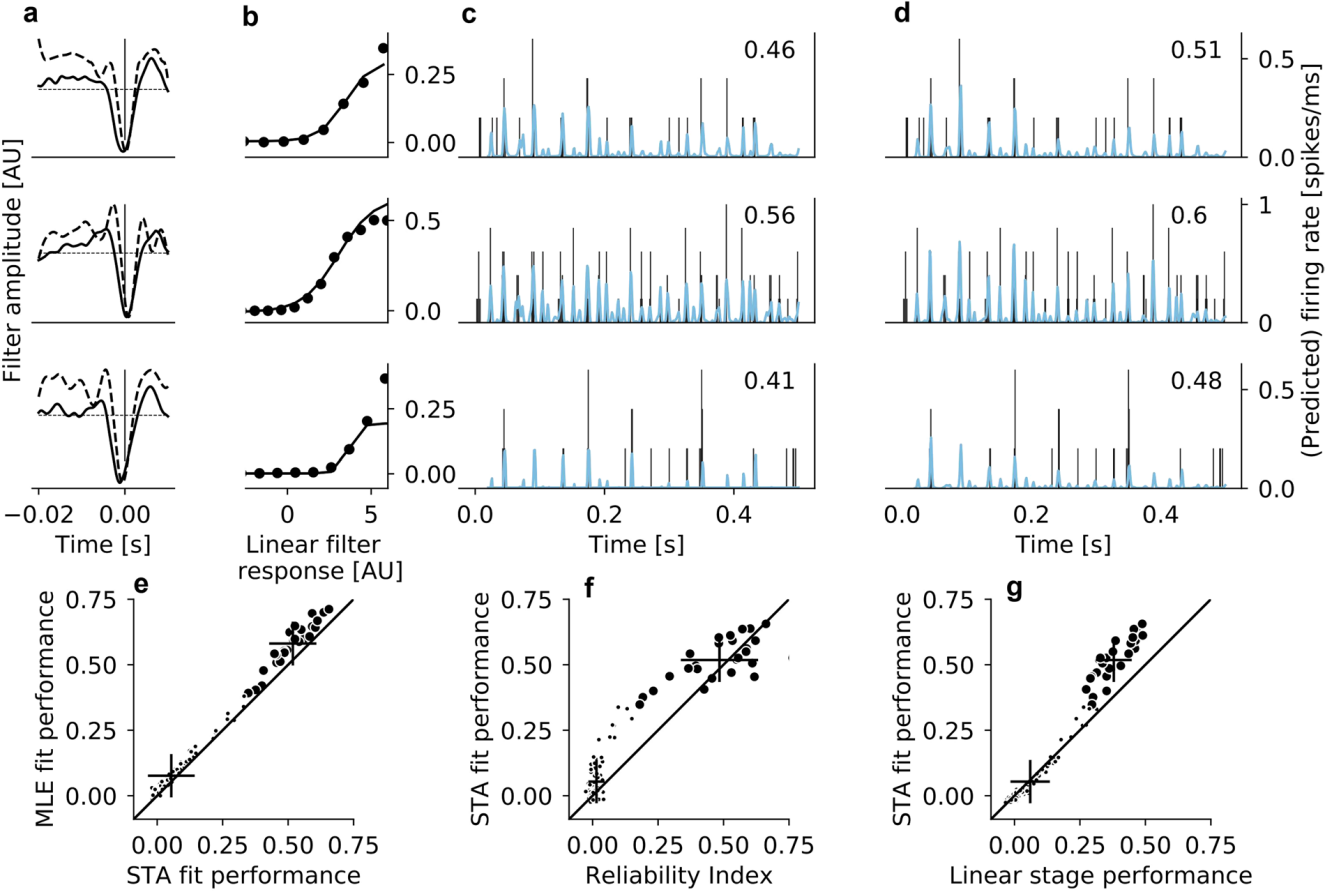
Model prediction of firing rates in *rd10* retina. (**a**) Linear filters of three example cells, derived from the STA fit (solid lines) and from the MLE fit (dashed lines); displayed at different scales in arbitrary units. To create the firing rate prediction, the filter response of the stimulus snippets with these filters was computed, and then the nonlinearity was applied. (**b**) Nonlinearity of the STA model; the nonlinearity of the MLE was the standard sigmoid function. (**c**) True firing rate, binned into 1 ms bins (black histogram) and the firing rate prediction of the STA fit of the model (blue traces) for three example cells. Decimal numbers in upper right corner indicate the performance of the models for that specific cell. (**d**) Same as (**c**) for the MLE fit of the model. (**e**) Prediction performance of the MLE fit plotted against the prediction performance of the STA fit. Small black dots represent RGCs that did not pass the reliability criterion of *RI* $> \,0.15$, but are shown here for completeness. (**f**) Prediction performance of the STA fit plotted against reliability index. Small black dots as in (**e**). (**g**) Prediction performance of the STA fit of the full LNP model plotted against the prediction performance of only the linear stage of the LNP model. Small black dots as in (**e**).

We predicted the firing rates of RGCs from *wt* and *rd10* mouse retina using the STA and the MLE fit of the LNP model (see Methods, Fig. 6a,b (*wt*); Fig. 7a,b (*rd10*)). The models were fit on 80% of the data (4 s of the stimulus), and the parameters recovered from this fit were used for the prediction of the remaining 20% of the data (1 s of stimulus). For RGCs from *wt* retina, prediction performance ${P}^{wt}$, evaluated as the correlation between true and predicted firing rate (Methods, Eq. (24)) ranged from 0.05 to 0.67 for the STA fit and from 0.1 to 0.7 for the MLE fit of the model (Fig. 6c,d). The MLE fit performed slightly better than the STA fit (mean ${\left\langle P\right\rangle }_{STA}^{wt}=0.46\pm 0.15$, mean ${\left\langle P\right\rangle }_{MLE}^{wt}=0.48\pm 0.15$), and both models performed better for cells with monophasic negative and biphasic filter shapes compared to cells with monophasic positive filter shapes (Table 2 and Fig. 6e).

**Table 2:** Prediction performance of the LNP model (STA and MLE fit) Prediction performance (mean $\pm$ s.d., [range]) of the STA fit and the MLE fit, measured as the correlation coefficient ${C}_{r\widehat{r}}$ (see Methods) between predicted and true firing rate at a resolution of 1 kHz, evaluated separately for the three identified clusters among *wt* filters, as well as for *rd10* filters.

	STA fit performance	MLE fit performance
monophasic negative (green, N = 34)	$0.52\pm 0.09,\hspace{2.22144pt}[0.32,0.67]$	$0.54\pm 0.09,\hspace{2.22144pt}[0.35,0.70]$
monophasic positive (red, N = 11)	$0.21\pm 0.11,\hspace{2.22144pt}[0.05,0.41]$	$0.25\pm 0.11,\hspace{2.22144pt}[0.10,0.42]$
biphasic (violet, N = 8)	$0.49\pm 0.06,\hspace{2.22144pt}[0.39,0.58]$	$0.54\pm 0.06,\hspace{2.22144pt}[0.45,0.63]$
*wt* all (N = 53)	$0.46\pm 0.15,\hspace{2.22144pt}[0.05,0.67]$	$0.48\pm 0.15,\hspace{2.22144pt}[0.10,0.70]$
*rd10* all (N = 26)	$0.52\pm 0.08,\hspace{2.22144pt}[0.35,0.66]$	$0.58\pm 0.09,\hspace{2.22144pt}[0.39,0.71]$

We hypothesized that there was a positive relationship between the prediction performance of the LNP model and the reliability of a cell’s response, indicated by its reliability index $RI$. Indeed, the performance of the model was generally better for more reliable cells (Fig. 6f). However, there were differences in model performance between cells with different filter shapes that could not be explained by differences in their response reliability; model performance was worse for cells with monophasic positive filters than for cells with monophasic negative filters, even when the cells were equally reliable in their response. Thus, the difference in model performance must either be due to differences in the quality of fit of the linear stage, the nonlinear stage, or a combination of both. To disentangle the effects of the linear and the nonlinear stage on model performance, we compared the accuracy of only the linear stage to that of the full model in predicting the firing rate. The linear stage of the LNP model can be used to predict the firing rate by computing the dot product between the linear filter and the stimulus, without applying the static nonlinearity. The prediction performance of the linear stage was then determined in analogy to the full model (see Methods). Differences in performance between RGCs with monophasic negative and biphasic filters on the one hand and RGCs with monophasic positive filters on the other hand already emerged at the linear stage (Fig. 6g, x-dimension). The prediction performance further diverged when applying the nonlinearity (Fig. 6g, y-dimension); compared to the linear prediction, the nonlinear prediction was significantly better for RGCs with monophasic negative filters (Wilcoxon signed-rank test, $p=3.67\times 1{0}^{-7}$, $\alpha =0.05$) and for RGCs with biphasic filters (Wilcoxon signed-rank test, $p=0.01$, $\alpha =0.05$), but not for RGCs with monophasic positive filters (Wilcoxon signed-rank test, $p=1$, $\alpha =0.05$). Thus, differences in model performance between cells are a cumulative effect of differences in the reliability of the cells’ response, but also of differences in the quality of the fit of the linear stage as well as the nonlinear stage.

Different filter shapes are the result of different response patterns that are elicited by the stimulus. Interestingly, both model fits also predict differences in response patterns between cells with different filter shapes (Fig. 6c,d, highlighted region). As noted before, linear filters of RGCs from *rd10* retina were most comparable to monophasic negative and biphasic filters of RGCs from *wt* retina (Fig. 3h,i,m and Fig. 7a). Likewise, patterns of model performance were similar in *rd10* RGCs and *wt* RGCs with monophasic negative and biphasic filters. For RGCs from photoreceptor-degenerated retina, the performance of the STA fit of the model ranged from 0.35 to 0.66 (${\langle P\rangle }_{STA}^{rd10}=0.52\pm 0.08$); again, the MLE fit performed slightly better (range 0.39 to 0.71, ${\langle P\rangle }_{MLE}^{rd10}=0.58\pm 0.09$, Fig. 7c–e). These performance values are very similar to those obtained for RGCs with monophasic negative filters from *wt* retina. Furthermore, the relationship between reliability of the response and LNP performance (Fig. 7f), as well as the relationship between linear stage performance and full model performance (Fig. 7g) in *rd10* retina were similar to these relationships for RGCs with monophasic negative and biphasic filters from *wt* retina (Fig. 6f,g).

## Discussion

Retinal implants represent a promising treatment option for patients suffering from degenerative retinal diseases like retinitis pigmentosa or macular degeneration, but clinical efficacy remains limited due to non-physiological and therefore suboptimal stimulation strategies. The goal of this study was to investigate the response properties of retinal ganglion cells to a novel electrical stimulus that comes closer to the physiological signals in the retina. Identifying an electrical stimulus that is both physiologically plausible and technically feasible might significantly improve clinical outcomes in patients wearing retinal implants.

We electrically stimulated retinal ganglion cells in wild-type and photoreceptor-degenerated mouse retina with smooth Gaussian white noise currents and used a model-based approach to estimate linear filters of the RGCs. The estimates of the linear filters could be clustered into three different groups based on their shapes and constituted the linear stage of a linear-nonlinear-Poisson model, which accurately predicted retinal ganglion cell firing probability. We demonstrate that physiologically plausible electrical stimuli can be used to activate retinal ganglion cells in both wild-type and photoreceptor-degenerated retina; furthermore, the LNP model could be used to find stimuli that maximize the response in cells with one class of filters while minimizing the response in cells with a different class of filter.

System identification approaches to estimate preferred stimuli of neurons are always limited to finding the preferred stimulus within the subspace that is spanned by the presented stimuli; if the true preferred stimulus is not in this subspace, it can only be approximated by the most closely matching stimulus within the subspace that was sampled^35,49^. Our smooth electrical stimulus broadly, but certainly not exhaustively, samples stimulus space, simultaneously probing different stimulus dimensions like frequency, amplitude and polarity. However, the stimulus is low-pass filtered at 100 Hz, posing a limitation on our sampling of frequency space; we cannot exclude that the true preferred electrical stimuli of RGCs are of higher frequency. The linear filters shown here should therefore be interpreted as the preferred electrical stimuli of RGCs within the subspace sampled by our stimulus.

One of the main goals in retinal prosthetics is the selective activation of different information channels in the retina, most prominently the ON and OFF channels. One way of achieving this goal are cell-type specific preferred electrical stimuli, like it is the case for light stimulation of healthy RGCs^43,47^. Indeed, investigating network-mediated electrical activation of rat RGC, ON cells were found to prefer anodic-first biphasic pulses, and OFF cells to prefer cathodic-first pulses, but the difference in preference was small^39^. Antagonistic polarities of electrical filters in wild-type mouse ON and OFF cells have been reported as well^41^; however, the clinical applicability of these filters as stimuli is limited as they most likely arose due to photoreceptor activation^49^.

In this study, we found three different classes of RGC filters in response to smooth electrical stimulation in wild-type, and one class of filter in photoreceptor-degenerated mouse retina. The most numerous of the three classes of RGC filters in *wt*, the monophasic negative filter, reflects RGC activation by cathodic currents. This is in agreement with previous findings showing that RGCs are preferentially activated by cathodic currents in epiretinal configuration, irrespective of whether the mode of activation is direct or through the network. Anodic currents also activate RGCs in epiretinal configuration, but at higher thresholds^23^. Indeed, less RGCs had monophasic positive filters reflecting activation by anodic currents, and these cells also responded less reliably to electrical stimulation (see Figs. 3k and 6e–g). This suggests that at the given stimulation intensity, RGCs with a preference for anodic currents were activated just above threshold. Considering the reported preference of OFF cells for cathodic-first pulses^39^, it is surprising that we find biphasic filters with opposite polarity (anodic-first) in OFF cells.

We investigated whether the different classes of electrical filters relate to light response profiles of RGCs in the healthy mouse retina. We found only few reliably responding ON cells, suggesting the conclusion that our stimulus more selectively activates OFF and ON-OFF cells. However, a large fraction of cells responding reliably to the electrical stimulus could not be identified in the light condition and could therefore not be characterized in terms of their light response profiles. As these reliably responding RGCs might by ON, ON-OFF or OFF, we can not conclude that our stimulus more selectively activates one or the other cell type. However, among the OFF cells, RGCs with sustained and transient light response preferred different shapes of electrical stimuli, suggesting that they could be selectively activated by stimuli derived from their respective filters.

While we found different classes of filters in *wt* retina, we found only one type of filter in *rd10* retina, which might cast doubt on the clinical relevance of our findings. Specifically, if our electrical stimulus activated RGCs in healthy retina via photoreceptors, and the different filter shapes were due to network mechanisms between photoreceptors and bipolar cells^41,50^, selective activation of different pathways would not be achievable in blind retina. Therefore, identifying which retinal elements in the vertical pathway (photoreceptors, bipolar cells or RGCs) are activated by our stimulus is highly relevant. One way of determining the mode of activation is by using pharmacological blockers^51^. We did not perform experiments with pharmacological agents, but the temporal and spatial properties of the RGC responses, i.e. latency and spatial spread, can still inform us about the origin of the response. Because our stimulus is time-continuous, we cannot directly compute the response latency as the time difference between stimulus delivery and response. Instead, we used the latencies of the filter peaks relative to the spike time as a proxy for response latency. The filter peak latencies (see Table 1) are much shorter than the response latencies $> 20\,ms$ usually reported for photoreceptor-mediated activation of ganglion cells in rabbit and rat retina. It is therefore unlikely that the RGC responses in *wt* retina were due to activation of photoreceptors. Rather, the latencies of both positive and negative monophasic filter peaks fall in the range usually reported for bipolar cell activation^23,28,51^. Also, *wt* RGCs with (negative or positive) monophasic filters responded within a radius of $\approx 200\,\mu m$ of stimulation electrodes, congruent with reports of the spatial extent of sensitivity to electrical stimulation in mouse retina upon network-mediated stimulation^44–46,51^. Spatial integration over presynaptic inputs might lead to indirect activation of RGCs at distances of up to $\approx 200\,\mu m$ from the stimulating electrodes. The comparably more shallow drop in current density outside of the active stimulation area at the level of the INL (relative to the level of the RGCs) (Fig. 1e) might facilitate this mode of activation at larger distances to the stimulating electrodes.

Conversely, the short latencies of the negative peak of biphasic filters in *wt* and monophasic filters in *rd10* retina suggest that these filters arose due to direct ganglion cell activation^16,22^. Also, RGCs with this type of linear filter were only found at a cell-electrode distance of 0 $\mu m$ (see Fig. 2f and 3l). A possible explanation for this finding is that these cells were activated directly and just above threshold, and that the stimulation intensity at the ganglion cell layer was too weak at larger cell-electrode distances to surpass this threshold (see Fig 1e). One factor that could lead to a more focal activation in *rd10* retina compared to *wt* retina might be previously reported elevated activation thresholds in *rd10* retina^52^. However, recent studies found a large variability in direct activation thresholds in degenerated retina^53^ and no clear difference in thresholds for direct and indirect activation between *wt* and *rd10* retina^45,54^. Taken together, the temporal properties of biphasic filters in *wt* retina and filters in *rd10* retina suggest that these were the result of direct retinal ganglion cell activation, while both monophasic filter shapes found in *wt* retina likely reflect RGC responses due to bipolar cell activation. It is therefore conceivable that different retinal elements are activated in *wt* and *rd10* retina by smooth electrical stimulation at the given intensity: bipolar and ganglion cells in wild-type retina, and only ganglion cells in photoreceptor-degenerated retina.

If *wt* RGCs with monophasic positive and negative filters were indeed activated through the network as hypothesized above, then the short latencies of filter peaks in this study would indicate an improvement in temporal precision of network-mediated electrical activation of RGCs by the use of smooth over pulsatile electrical stimulation. Epiretinal stimulation of wild-type mouse retina with subthreshold pulsatile Gaussian white noise yielded biphasic filters with peak latencies roughly 2 orders of magnitude longer than the filters described here^40,41^. Filters estimated from network-mediated rat RGC responses to photovoltaic stimulation were about 10 times slower^50^. Comparing to direct estimates (as opposed to the proxy of filter peak latencies) of network-mediated response latencies found using pulsatile stimulation, the peak latencies of filters reported here are roughly 2–5 times shorter and less variable^39,55,56^.

Our stimulus could prove clinically relevant in several different ways. First, it could be used to directly activate individual RGCs in a spatially confined manner, avoiding axonal stimulation which causes the occurrence of elongated phosphenes experienced by patients wearing a retinal implant^57^. In this case, however, differential activation of retinal pathways could probably not be achieved by using selective stimulus waveforms, but would have to occur through targeted stimulation of identified RGCs^22,58,59^. Second, the short and precise latencies found here are a result of fast and precise RGC responses to electrical stimulation. Thus, a stimulus derived from the filters described here, implemented in a retinal prosthesis, might confer greater control over temporal activation patterns and thus help to overcome problems such as fading^18^.

Finally, our model predicts differences in electrically evoked responses of cells with different filter shapes (see Fig. 6c,d, highlighted region) and might therefore be used to identify stimuli that will maximize the response of cells with one type of filter while minimizing the response in cells with a different type of filter^60^. Identifying such stimuli might potentially provide a tool for selective activation of certain cell types. However, different filter shapes were so far only identified in wild-type retina, likely upon network-mediated activation. One factor affecting the possibility to activate RGCs via the network is the degree of degeneration of the retina. In this study we report findings from *rd10* retina at different advanced stages of degeneration (mice aged 80 to 209 days). In earlier stages of degeneration, however, the network remains intact in the *rd10* model of retinal degeneration as well as in patients suffering from retinitis pigmentosa^44,61–63^. Future studies should therefore investigate how the degree of degeneration affects RGC responses to our stimulus, and whether remaining intact circuitry can indirectly activate RGCs in degenerating retina upon smooth electrical stimulation.

In conclusion, future work will reveal to what extent the stimulus and modelling approach presented here will improve stimulation protocols for retinal prostheses in terms of spatial and temporal resolution and cell-type specificity of the stimulation.

## Methods

All experimental procedures were carried out in compliance with §4 of the German law on animal protection and were approved by the Regierungspräsidium Tübingen (Registration No.: 35/9185.82-7). All the experiments were performed in accordance with the ARVO statement for the use of animals in ophthalmic and visual research.

### Retina preparation

*Ex vivo* retina from five B6.CXB1-Pde6brd10/J (*rd10*) mice (2 females, 3 males; age between post-natal days 80 and 209 (p80 and p209) and from three C57BL/6J (*wt*) mice (2 female, 1 male; age between p87 and p274) was used. Animals were dark-adapted for approximately 30 minutes before the experiment, anaesthetised with carbondioxide and euthanized by cervical dislocation. Both eyes were then enucleated, the retina was isolated and dissected in Ames’ buffer under dim red-light conditions to prevent bleaching of remaining photoreceptors (further details have been described previously^44,64^). A retinal portion of ca. 1-2 $m{m}^{2}$ was placed on a CMOS-based microelectrode array in epiretinal flat-mount configuration. Before each use, the microchip was cleaned with Terg-a-zyme (Sigma Aldrich, Z273287 dissolved in bidistilled water) and then coated with Poly-L-lysine (Sigma Aldrich, P2636, 1 mg/ml dissolved in bidistilled water). The recording chamber (2 *ml*) was perfused with warm, carbonated Ames’ buffer (Sigma Aldrich, A1420, 36 ${}^{\circ }$C, pH 7.4) at a flow rate of $4\frac{ml}{min}$.

### High-density CMOS-based microelectrode array (hd CMOS-MEA)

Stimulation and recording of retinal ganglion cell activity were performed with a high-density CMOS-based microelectrode array (CMOS MEA 5000, Multi Channel Systems MCS GmbH, Reutlingen, Germany). The MEA comprised 1024 capacitive stimulation electrodes and 4225 recording electrodes on an area of $1\,m{m}^{2}$ (see Fig. 1). Each stimulation electrode extended over an area of 688 $\mu {m}^{2}$ with 0.5 $\mu m$ spacing between stimulation electrodes. The stimulation electrodes were made of a titanium nitrite and were covered by a native oxide layer which insulated the metal from the electrolyte in the recording chamber. The pitch (center-to-center spacing) between two recording electrodes was 16 $\mu m$. The stimulation software (https://www.multichannelsystems.com/products/cmos-mea-control) allowed to define arbitrary stimulation areas by choosing a stimulation electrode to be active or inactive independently for each stimulation site (see Fig. 1a). The resolution of the Digital-to-Analog Converter was 16 bit. Details of the CMOS-based MEA have been reported previously^65,66^.

### Electrical stimulation

We designed an electrical stimulus consisting of Gaussian white noise low-pass filtered at 100 Hz with a ${5}^{th}$ order Butterworth filter (Fig. 1b). The electrical stimulus was applied using the hd CMOS-MEA 5000 (see Fig. 1a). Applying voltages to the stimulation electrodes of the chip evokes capacitive currents in the electrolyte. The stimulation current density is proportional to the time derivative of the electrode voltage and scales with the specific electrode capacitance *c*:

1 $${i}_{stim}=c\frac{dV}{dt}$$

**Figure 8:**
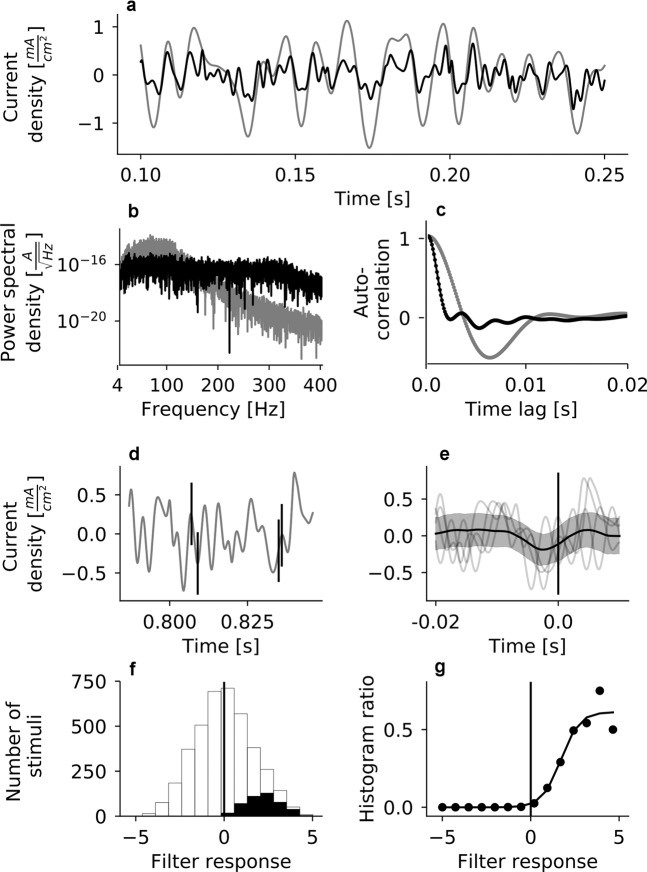
LNP model: Stimulus whitening and filter estimation by spike-triggered averaging. (**a**) Example segment of the original stimulus in grey, and the corresponding segment of the whitened stimulus in black. (**b**) Power spectral density (PSD) of the original (grey) and whitened (black) stimulus. While the PSD of the original stimulus declines at frequencies $>$100 Hz due to filtering, this effect is undone by whitening. (**c**) The autocorrelation function of the original (grey) and the whitened (black) stimulus at time lags of up to $20$ ms. The temporal extent of the autocorrelation of the whitened stimulus is much reduced compared to the original. (**d**) For every spike of a RGC (black vertical lines), a segment of the whitened version of the stimulus, spanning 20 ms before and 10 ms after the spike, is added to the spike-triggered ensemble (STE). (**e**) The spike-triggered average (black trace) is computed by averaging across all elements of the STE (the STE elements from panel (a) are shown as thin gray lines). Gray shaded area indicates $\pm 1$ standard deviation of the STE. Vertical black line indicates time of spike. (**f**) The elements of the full stimulus ensemble, consisting of all 300 sample long stimulus snippets taken 10 samples apart, are projected onto the linear filter of a cell and binned to yield a histogram (empty bars). The same is done for the elements of the STE (black histogram). (**g**) The nonlinearity (black dots) is estimated as the ratio between the histogram of the projected elements of the spike-triggered ensemble to the histogram of the projected elements of the full stimulus ensemble (black and the empty histograms from panel (**f**), respectively) and fit by a sigmoidal (Eq. (14)) or exponential (Eq. (15)) function (black trace, here sigmoidal).

Therefore, in order to obtain a certain current $${i}_{stim}$$, the integral function of the desired current has to be applied to the chip as voltage command. For our electrical Gaussian white noise stimulus, this was achieved by generating $${f}_{s}\cdot T$$ samples (where $${f}_{s}$$ is the sampling frequency and $$t$$ is the total stimulus duration) from a standard normal distribution, and then calculating the cumulative sum over these samples. To reduce the drift in the random walk that is generated by this process, a reflection limit $${L}_{reflect}$$ was introduced: whenever adding a new sample to the cumulative sum would raise the absolute value of the cumulative sum above the reflection limit $${L}_{reflect}=10$$, the step’s sign was inverted. This yielded a sequence of values (in arbitrary units) describing a random walk, which was then low-pass filtered at 100 Hz with a 5^th^ order Butterworth filter and rescaled to generate a voltage command $${V}_{command}$$ that respects the safe input limits of the stimulus generator, $$[0V\le {V}_{command}\le 2.5V]$$. The introduction of the reflection limit reduces the power of the stimulus in the low frequency regime; apart from that, power spectral density of the resulting signal is relatively flat up to a frequency of 100 Hz and then drops off (Fig. 8b; note that frequencies $$ < 4$$ Hz are not shown to improve visibility).

The stimulus applied in the experiments described here was generated using the parameters ${f}_{s}=10$ kHz, $T=5\ s$ and ${L}_{reflect}=10\ (AU)$. The stimulation current was measured as described before^45,55^. The maximal current density achieved with these parameters and the available chips was ${i}_{max}\approx 1.5\ \frac{mA}{c{m}^{2}}$. Absolute current magnitude depended on the area of the active stimulation sites; for most recordings a subset of the stimulation electrodes was chosen (see Fig. 1a), while for one recording of *rd10* retina, the whole array was stimulated. These conditions are referred to as subfield and fullfield condition, respectively. Active electrodes in the subfield condition were chosen such as to cover areas with good contact between retina and CMOS-MEA as reflected by RGC activity detected on the recording electrodes.The same Gaussian white noise stimulus was played on all active electrodes throughout all conditions and experiments. We pool across conditions and set the distance of RGCs to stimulation electrode to 0 for all cells in the fullfield condition.

### Simulation of current density

We simulated the current density in the retina with the finite-element method using the software COMSOL Multiphysics ®. We modeled the retina as a cylinder with a radius of 1 $mm$ and varied its height to simulate *wt* retina (height of 200 $\mu m$) and *rd10* retina (height of 100 $\mu m$). All other parameters were identical in the simulations for *wt* and *rd10* retina. A second cylinder with the same radius and a height of 1 $mm$ was stacked on top to model the surrounding electrolyte. On the bottom of the retinal cylinder we centrally placed 5 $\times$ 5 or 32 $\times$ 32 electrodes to simulate subfield and fullfield stimulation conditions, respectively. The stimulation electrodes were modeled to be flat with shapes identical to the experimentally used electrodes (see Fig. 1a, inset). The comparably large and remote reference electrode was modeled by setting the top of the electrolyte cylinder to an electrical potential of zero. For the stimulation electrodes we assumed a homogeneous surface current density of 1.5 $\frac{mA}{c{m}^{2}}$. All other boundaries were assumed to be perfect insulators with a normal current density of 0. The electrical resistivities of the retina and the electrolyte were assumed to be homogeneous and isotropic with values of 1000$\Omega \cdot cm$ and 65$\Omega \cdot cm$, respectively^16^. We modeled rectangular stimulation areas of different sizes (5 $\times$ 5 and 32 $\times$ 32) and computed the norm of the current density for different lateral distances to the center of the stimulation area between 0 and 400 $\mu m$ in steps of 0.2 $um$. Due to the shapes and placements of the electrodes on the chip, the current density at a fixed lateral distance varies with angle. Therefore, we also computed the current density for each lateral distance at angles between 0 and 90${}^{\circ }$ in steps of 0.2${}^{\circ }$. The current density was then averaged across all angles. This was done for a vertical distance to the stimulation electrodes (height) of 20 $\mu m$ (approximately ganglion cell layer^67,68^) and 90 $\mu m$ (approximately inner nuclear layer) (see Fig. 1e,f). For further details on the simulation, please see Oesterle *et al*.^69^.

### Light stimulation

To probe the light response of RGCs in wild-type retina, we presented full-field light flashes repeated 4 to 10 times. Light stimuli were generated by a selected LED (pE 4000 coolLED, peak wavelength 470–490 $nm$) and were flashed on for 500 ms, then turned off for 500 ms, thus creating a 1 Hz flicker. The light stimuli were projected onto a custom-made digital mirror display ($\mu$-Matrix, Rapp Optoelectronic GmbH) mounted on an upright microscope (Olympus, BX51WI). The digital mirror display was focused onto the backside of the microscope objective.

### Light response analysis

The light response profile of RGCs from *wt* retina was quantified by calculating a Bias Index (BI) and a Transiency Index (TI)^43^. The Bias Index is calculated as

2 $$BI=\frac{{R}_{ON}-{R}_{OFF}}{{R}_{ON}+{R}_{OFF}}$$

where ${R}_{ON}$ and ${R}_{OFF}$ are the total number of spikes fired by a cell in the first 300 ms after light onset and light offset, respectively, summed across all repetitions. The Bias Index ranges from -1 for OFF cells through 0 for ON-OFF cells to 1 for ON cells. RGCs with $BI < -0.25$ are termed OFF cells, RGCs with $-0.25\le BI\le 0.5$ are termed ON-OFF cells and RGCs with $BI > 0.5$ are termed ON cells. The thresholds were chosen based on the distribution of BIs in the RGC population recorded here. The Transiency Index is defined as

3 $$1-\left\langle {R}_{pref}\right\rangle $$

where $\langle {R}_{pref}\rangle$ denotes the average normalized histogram (binsize 50 ms) in the 500 ms window following a cell’s preferred light stimulus (light onset or light offset). TI ranges from 0 (sustained; maximum firing rate maintained throughout duration of preferred stimulus) to $1-\frac{1}{numberofbins}$ (transient; only fires in the first bin after stimulus onset; here $1-\frac{1}{10}=0.9$). For ON-OFF cells, the TI is defined as the mean between the TI for the OFF phase of the stimulus and the TI for the ON phase of the stimulus.

### Artefact reduction and preprocessing

In the raw recording, spiking activity is masked by large artefacts caused by the stimulation current, making spike sorting and further analysis impossible (Fig. 1b,c). Therefore, a series of data preprocessing steps was performed to remove the stimulation artefact. First, the raw traces of each recording electrode were band-pass filtered between 1000 and 9500 Hz with a ${2}^{nd}$ order Bessel filter. Then, because the time courses of the stimulation current and the artefact recorded on the sensors are aligned^70^, the contribution of the stimulation artefact to the raw trace could be estimated by computing the dot product between the applied stimulus $S$ and the raw trace ${r}_{raw}$ (${r}_{raw},S$ are N-dimensional column vectors), normalized to the dot product of the stimulation waveform with itself:

4 $$f=\frac{{r}_{raw}^{\top }S}{{S}^{\top }S}.$$

To remove the contribution of the stimulation artefact in the raw recording, the stimulus trace (Fig. 1b) was then subtracted from the raw recording (Fig. 1c), multiplied by the factor $f$:

5 $${r}_{pre}={r}_{raw}-f\cdot S.$$

In the resulting signal, spikes are clearly separated from the background signal and no longer masked by the stimulation artefact (Fig. 1d). Spike sorting was performed on this signal using the spike sorter based on convolutive independent component analysis^71^, implemented in the MultiChannel Systems CMOS-MEA-Tools Software (https://www.multichannelsystems.com/software/cmos-mea-tools, version 2.1.0).

### Evaluating reliability of RGC responses to electrical stimulation

In order to quantify how reliably RGCs respond across repetitions of the same stimulus, we computed a *Reliability Index* (*RI*). To compute the *RI*, individual RGC responses from 4 repetitions were binned into $2\hspace{2.22144pt}ms$ wide bins, resulting in a histogram *H*. The same was done for the remaining ${5}^{th}$ repetition, yielding a histogram *h*. Then the correlation coefficient between the two histograms was computed. This was repeated for all possible combinations of *H* and *h*, and the *RI* was computed as the average across the resulting correlation coefficients:

6 $$RI=\left\langle \frac{{C}_{Hh}}{\sqrt{{C}_{HH}{C}_{hh}}}\right\rangle ,$$

where $C$ denotes the covariance matrix of the histogram $h$ of cell responses during four repetitions of the stimulus and the histogram $h$ of the cell responses during the fifth repetition, and angular brackets $\langle \rangle$ denote the mean across all (five) possible combinations of $H$ and $h$. A value of 1 indicates perfect reliability (identical response pattern in all repetitions), while a value close to 0 indicates no reliability in the response to the stimulus. Based on visual inspection of the distribution of the RIs of wild-type RGCs, we included RGCs in the analysis if their RI was larger than $0.15$ (see Fig. 2d). This threshold was compatible with the distribution of RIs of RGCs from *rd10* retina (see Fig. 2g).

### Fitting a linear-nonlinear-Poisson model to electrically evoked RGC responses

We modeled RGC responses to electrical stimulation with a Linear-nonlinear-Poisson (LNP) model^35^. The LNP model assumes that the firing rate of a neuron in response to a stimulus ${s}_{t}$ at time $t$ can be modelled as an inhomogeneous Poisson process with instantaneous firing rate

7 $${\mu }_{t}={\mathcal{N}}({{\boldsymbol{k}}}^{\top }{{\boldsymbol{s}}}_{t})$$

where ${{\boldsymbol{k}}}^{\top }{{\boldsymbol{s}}}_{t}$ denotes the dot product of the cells linear filter and the stimulus at time t, and ${\mathcal{N}}$ denotes a static nonlinearity.

We used two different approaches to fit the LNP, spike-triggered averaging with subsequent estimation of the nonlinearity, and maximum-likelihood estimation of a generalized linear model (GLM) with logit link function.

#### LNP model: Filter estimation by spike-triggered averaging

The spike-triggered average^34,72^ (STA) is calculated by averaging over the spike-triggered stimulus ensemble (STE), which is the collection of all stimuli that were followed by a spike within a defined time window (here $1\,ms$). For a discrete time shift ${\tau }_{i}$ before the spike the STA is given as

8 $$v({\tau }_{i})=\frac{1}{n}\mathop{\sum }\limits_{k=1}^{n}s({t}_{k}-{\tau }_{i}),$$

with

$N$ the number of spikes, $s(t)$ the value of the stimulus at time $t$, ${\tau }_{i}$ the time shift relative to the spike.

A cell’s linear temporal filter is then defined as the vector

9 $${\boldsymbol{v}}=(v({\tau }_{max}),v({\tau }_{n-1}),\ldots ,v({\tau }_{0}))$$

The spike-triggered average was calculated 20 ms into the past and 10 ms into the “future” relative to the spike, i.e. ${\tau }_{max}=-20$ ms and ${\tau }_{0}=10$ ms. The duration of the filters presented here is thus 30 ms, equivalent to 300 stimulus samples.

The stimulus was low-pass filtered at 100 Hz (see Methods) and therefore has a non-zero autocorrelation at time shifts $\le 10\hspace{2.22144pt}ms$ (see Fig. 8c). These autocorrelations affect the shape of the filters computed by spike-triggered averaging^73^. To reduce the effect of the autocorrelations on the filters, the STA was calculated using whitened stimulus "snippets”. For the whitening procedure^74^, the 50 000 samples (5 s) long continuous stimulus was sliced into snippets of 300 stimulus samples, taken 10 samples apart (1 ms). This yielded a stimulus ensemble containing 4701 stimulus snippets, and the covariance matrix was computed based on this ensemble. The square-root of the pseudo-inverse of the resulting covariance matrix, ${C}_{stim}^{-\frac{1}{2}}$, was computed, and each stimulus snippet was whitened by multiplying with ${C}_{stim}^{-\frac{1}{2}}$:

10 $${{\boldsymbol{s}}}_{i}^{white}={C}_{stim}^{-\frac{1}{2}}{{\boldsymbol{s}}}_{i}$$

with

${{\boldsymbol{s}}}_{i}\in {{\mathbb{R}}}^{300\times 1}$ stimulus “snippets” of 300 frames each ${C}_{stim}^{-\frac{1}{2}}\in {{\mathbb{R}}}^{300\times 300}$ the pseudoinverse of the stimulus covariance matrix ${C}_{stim}$.

The pseudoinverse is obtained from the eigendecomposition of the stimulus covariance matrix

11 $${C}_{stim}=\mathop{\sum }\limits_{i=1}^{K\le M}{\lambda }_{i}{{\boldsymbol{e}}}_{i}{{\boldsymbol{e}}}_{i}^{\top }$$

by keeping the eigenvectors ${{\boldsymbol{e}}}_{i}\in {{\mathbb{R}}}^{300\times 1}$, but inverting and taking the square root of the $L < K$ largest eigenvalues ${\lambda }_{i}$ and setting the remaining eigenvalues to zero:

12 $${C}_{stim}^{-\frac{1}{2}}=\mathop{\sum }\limits_{i=1}^{L}{\lambda }_{i}^{-\frac{1}{2}}{{\boldsymbol{e}}}_{i}{{\boldsymbol{e}}}_{i}^{\top }$$

The whitened stimulus used for spike-triggered averaging (Fig. 8a,d) was reconstructed by concatenating the resulting whitened stimulus snippets and smoothing the edges with a hanning window.

The nonlinearity ${\mathcal{F}}$ was estimated as the ratio between the histograms of the projection of the STE onto the cells linear filter and the projection of the raw stimulus ensemble onto the cells linear filter^35^ (Fig. 8f,g):

13 $$\widehat{{\mathcal{F}}}=\frac{{H}_{STE}}{{H}_{FE}}$$

${H}_{STE}$ denotes the histogram of projected stimulus snippets from the spike-triggered ensemble (STE) and ${H}_{FE}$ denotes the histogram of projected stimulus snippets from the full ensemble (FE). The estimate of the nonlinearity was then fit by a sigmoidal or exponential function. The sigmoidal fit to the nonlinearity at time $t$ is described by the equation

14 $${\widehat{{\mathcal{F}}}}_{sig}({{\boldsymbol{v}}}^{\top }{{\boldsymbol{s}}}_{t})=\frac{{y}_{max}}{1+\exp (-g({{\boldsymbol{v}}}^{\top }{s}_{t}-{x}_{0}))}+{y}_{min}$$

with free parameters ${y}_{max}$ (saturation amplitude of the sigmoidal curve), $g$ (gain factor), ${x}_{0}$ (50% threshold of the saturation amplitude) and ${y}_{min}$ (vertical offset). The exponential fit to the nonlinearity at time $t$ is described by the equation

15 $${\widehat{{\mathcal{F}}}}_{exp}({{\boldsymbol{v}}}^{\top }{{\boldsymbol{s}}}_{t})=a\exp (g({{\boldsymbol{v}}}^{\top }{s}_{t}))+{y}_{min}$$

with free parameters $a$ (controlling the height of the curve), $g$ (gain factor), and ${y}_{min}$ (vertical offset). ${s}_{t}$ is a whitened stimulus snippet as defined in Eq. (10), containing the 300 stimulus samples preceding $t$.

#### LNP model: Filter estimation by maximum-likelihood estimation

In addition to the spike-triggered approach, the temporal electrical filter was also estimated by fitting a GLM with logit link function and an elastic net penalty to the data^75,76^. Here, the filter coefficients are found by minimizing the negative log-likelihood of the observed data with respect to the coefficients. To constrain the coefficients and to prevent overfitting, a penalty term is added to the loss function. The GLM was fit on the raw (not whitened) stimulus, because the process of maximum likelihood estimation intrinsically performs correlation-correction on the filter estimate^77^. We used the scikit-learn python package^78^ for fitting the GLM and for cross-validation.

The firing probability ${\mu }_{t}$ at time $t$ is assumed to be related to the stimulus by a sigmoid function

16 $${\mu }_{t}=\frac{1}{1+\exp (-{z}_{t})}$$

where

17 $${z}_{t}={\beta }_{0}+\sum _{j}{\beta }_{j}{s}_{tj}$$

with

${\beta }_{0}\in {\mathbb{R}}$ a scalar offset $\beta \in {{\mathbb{R}}}^{m}$ the estimate of the m-dimensional linear filter

is a linear transformation of the stimulus ${{\boldsymbol{s}}}_{t}\in {{\mathbb{R}}}^{300\times 1}$ at time $t$. Assuming that a spike train $Y=\{{y}_{1},{y}_{2},...,{y}_{t}\}$, ${y}_{i}\in \{0,1\}$ of one particular neuron is generated by a sequence of independent (but not identically distributed) Bernoulli trials with time-dependent firing probability ${\mu }_{t}$, the probability of the spike train is given by

18 $$P(Y| {\mu }_{1},{\mu }_{2},\ldots ,{\mu }_{t})=\prod _{t}{{\mu }_{t}}^{{y}_{t}}{(1-{\mu }_{t})}^{(1-{y}_{t})}$$

The average log-likelihood function ${\mathcal{L}}({\boldsymbol{\mu }};Y)$ of the parameters ${\boldsymbol{\mu }}$ given the observed spike train $Y$ is obtained by taking the logarithm of equation (18) and dividing by the number of spikes $N$:

19 $${\mathcal{L}}({\boldsymbol{\mu }};Y)=\frac{1}{N}\sum _{t}{y}_{t}{\rm{\log }}\,({\mu }_{t})+(1-{y}_{t}){\rm{\log }}\,(1-{\mu }_{t})$$

Adding the elastic net penalty,

20 $$\lambda [(1-\alpha ){P}_{2}+\alpha {P}_{1}]$$

with

21 $${P}_{1}=\sum _{j}{\beta }_{j}$$

22 $${P}_{2}=\frac{1}{2}\sum _{j}{\beta }_{j}^{2}$$

to the average negative log-likelihood function (Eq. (19)) yields the loss function

23 $${\mathcal{J}}=-\frac{1}{N}\sum _{t}{y}_{t}{\rm{\log }}\,({\mu }_{t})+(1-{y}_{t}){\rm{\log }}\,(1-{\mu }_{t})+\lambda \left[(1-\alpha )\frac{1}{2}{P}_{2}+\alpha {P}_{1}\right]$$

The values of the parameters ${\beta }_{0}$ and ${\boldsymbol{\beta }}$ which minimize the loss function were found by using the SAGA solver implemented in the scikit-learn package. The resulting ${\boldsymbol{\beta }}$ represents the linear filter of the LNP model estimated by maximum likelihood estimation (MLE). The hyperparameters $\lambda$ (determining the strength of the regularization) and $\alpha \in [0,1]$ (determining the relative contributions of the L1 (Eq. (21)) and L2 (Eq. (22)) regularization terms) were determined by a cross-validated parameter grid search, choosing $\lambda$ from 5 logarithmically spaced values from the interval $[0.0001,0.01]$, and $\alpha$ from $\{0,0.01,0.1,0.5,1\}$.

#### Model prediction and evaluation

Both the STA fit and the MLE fit of the LNP were used to predict RGC spiking activity in response to the stimulus: the filter response of the stimulus was computed, and the nonlinearity was applied to the filter response, yielding the firing rate prediction. To prevent overfitting and overestimation of the model prediction performance, the model was fit on four seconds of stimulus, and the parameters derived from that fit were used to predict the firing rate in the remaining leftout second of the stimulus. This was done for all five possible splits into four training seconds and one test second.

Prediction performance $P$ was evaluated as the correlation coefficient between true ($r$) and predicted firing rate ($\widehat{r}$) at a resolution of 1 kHz:

24 $$P=\frac{{C}_{{\boldsymbol{r}}\widehat{{\boldsymbol{r}}}}}{{\sigma }_{{\boldsymbol{r}}}{\sigma }_{\widehat{{\boldsymbol{r}}}}}$$

where $C$ denotes the covariance matrix of the true and the estimated firing rates $r$ and $\widehat{r}$.

### Hierarchical clustering

A hierarchical clustering algorithm was used to cluster the filters of all cells to determine whether there were different classes of filters. At first, the dimensionality of the filters was reduced from $N=300$ features (30 ms at a sampling rate of 10 kHz) to $k < N$ features retaining 90% of the variance by applying principal component analysis (PCA) and projecting the filters onto the retained $k$ principal components (PCs). Hierarchical clustering was then performed on these projections, using the scipy.cluster.hierarchy package with the average euclidean distance in PC space as clustering metric. The number of clusters was determined by visual inspection of the resulting dendrogram.

## Supplementary information

Supplementary Information.

## Data Availability

The datasets generated and analysed during the current study, as well as the code used for analysis, are available from the corresponding author on reasonable request.
