# Supplementary material for: Probing and predicting ganglion cell responses to smooth electrical stimulation in healthy and blind mouse retina
Source: Sci Rep. 2020 Mar 23;10:5248. doi: 10.1038/s41598-020-61899-y (PMC7090015; doi:10.1038/s41598-020-61899-y)
Supplement: Supplementary file 1 — Supplementary Information. [file 41598_2020_61899_MOESM1_ESM.pdf]

Probing and predicting ganglion cell responses to smooth electrical stimulation in healthy and blind mouse retina

Larissa Höfling[1, 2, 3, 4], Jonathan Oesterle[3], Philipp Berens[3,5,6], and Günther Zeck[1,5,*]

[1]Natural and Medical Sciences Institute at the University of Tübingen, Reutlingen, Germany
[2]Graduate Training Centre of Neuroscience, University of Tübingen, Tübingen, Germany
[3]Institute for Ophthalmic Research, University of Tübingen, Tübingen, Germany
[4]Center for Integrative Neuroscience, University of Tübingen, Tübingen, Germany
[5]Bernstein Center for Computational Neuroscience, University of Tübingen, Tübingen, Germany
[6]Department of Computer Science, University of Tübingen, Tübingen, Germany
[*]Corresponding author: Guenther.Zeck@nmi.de

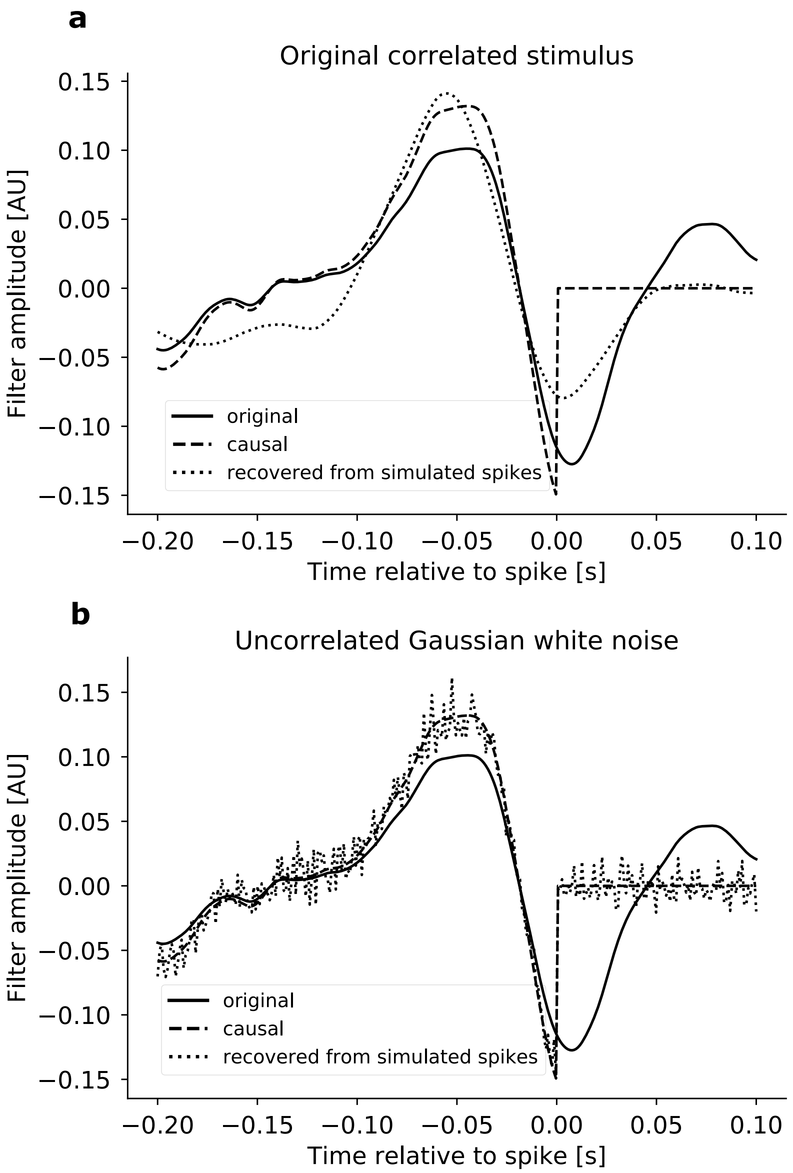

Figure S1. Stimulus correlations introduce acausal components in estimate of causal underlying filter We took an original filter from a real retinal neuron that contained acausal components, i.e. non-zero elements at times $t > t_{spike}$ (solid line in (a) and (b) and made it causal by setting these elements at $t > t_{spike}$ to 0 (dashed line in (a) and (b)). We then simulated spikes using this causal filter and the parameters of the nonlinearity obtained from the fit to the original cell's responses. We did this for two different stimuli: (a) the original, low-pass filtered and hence correlated stimulus used in the experiments; (b) and an unfiltered Gaussian white noise stimulus containing no correlations. We then estimated the filter again from these simulated spikes by STA (dotted line in (a) and (b)). While the filter recovered from simulated spikes in response to the original correlated stimulus shows acausal components (a), the filter recovered from simulated spikes in response to the uncorrelated stimulus is causal (b). This shows that stimulus correlations can lead to acausal components in filter estimates, even if the underlying filter that produced the response is causal, as we expect it to be the case in real neurons. All filters $\vec{w}$ shown are normed s. t. $||\vec{w}||_2 = 1$.
